# An outbreak of nosocomial infection of neonatal aseptic meningitis caused by echovirus 18

**DOI:** 10.1017/S0950268823000973

**Published:** 2023-06-14

**Authors:** Hongmin Xi, Yi Tian, Hui Shao, Xiangyun Yin, Lili Ma, Ping Yang, Xianghong Li

**Affiliations:** 1 Neonatology Department, The Affiliated Hospital of Qingdao University, Qingdao, China; 2 Jining Medical University, Jining, China

**Keywords:** enterovirus, neonate, nosocomial infection, outbreak, echovirus, meningitis

## Abstract

We describe an outbreak of echovirus 18 infection involving 10 patients in our neonatal intensive care unit (an attack rate of 33%). The mean age at the onset of illness was 26.8 days. Eighty percent were preterm infants. All were discharged home without sequelae. There were no differences in gestation age, birth weight, delivery mode, use of antibiotics, and parenteral nutrition between the enterovirus (EV) group and non-EV group, but the rate of breastfeeding was significantly higher in the EV group. Separation care and reinforcement of hand-washing seemed to be effective in preventing further spread of the virus. Visiting policy, hygiene practice, and handling of expressed breastmilk should be reinforced.

Enterovirus (EV) infection, a common pathogen that causes an outbreak of nosocomial infections in neonatal intensive care units (NICUs), often occurs in summer and autumn, accounting for 15.6% of neonatal nosocomial infections caused by viruses. EV has been reported to be one of the top viruses to cause outbreaks of nosocomial infections in NICUs in the United States every year [1]. Among them, echovirus and Coxsackie virus are common types involving high neonatal mortality, especially in early-onset infections [2]. An EV infection can be acquired either via vertical transmission from mother to child or by horizontal transmission in the NICU. However, there are few reports of echovirus 18 infections in neonates. An outbreak of echovirus 18 infection in 20 neonates was reported in Japan in 2007 [3]. In the present study, we report echovirus 18 infection outbreak in 10 neonates with aseptic meningitis.

In July 2019, sepsis-like manifestations were observed in 10 neonates in the NICU of our hospital. At that time, our hospital had a Grade III NICU with 31 patients admitted. On 14 July, the conditions of three hospitalised preterm infants declined, including of two infants (40 days after birth) who were twins (cases 1, 2) born at a gestational age (GA) of 29 weeks and one infant born at a GA of 30+5 weeks (20 days after birth, case 3), all having received full enteral feeding without any intravenous medication. Another preterm infant at a GA of 29+3 weeks (case 4) was re-admitted due to fever after 3 days of discharge. From 15 July to 19 July, six new cases were reported.

There were two term infants and eight preterm infants. The mean GA was 31.7±4.0 weeks, and the mean birth weight was 1812.9±1025.1 g. The term infants showed significantly elevated body temperature, without anorexia or diarrhoea. However, one term infant developed rashes. Most preterm infants had mild fever or normal body temperature, apnoea, unstable oxygenation, and feeding intolerance, without diarrhoea or vomiting; two preterm infants developed rashes. Two preterm infants had early manifestations of shock such as tachycardia and poor peripheral circulation. One preterm infant had bloody stools. Convulsion, disturbance of consciousness, and involvement of limb movement were not observed (Table 1). One infant had mild liver function impairment. Most patients experienced a mild increase in C-reactive protein (maximum 45.96 mg/dL) and normal procalcitonin (<0.5, except for one with 4.44 ng/ml). Cerebrospinal fluid (CSF) cell count was higher in one case. Cranial MRI for five preterm infants before discharge showed no abnormalities. Test for EV was performed on the CSF and stool by real-time polymerase chain reaction method in 10 patients. EV RNA was detected in the CSF and stool, and next-generation sequencing demonstrated an echovirus 18 outbreak. The results of bacterial culturing were negative in the initially collected samples, except for one of the expressed breastmilk samples, in which staphylococcus epidermidis developed. No severe lung parenchymal lesions were visible on chest X-ray. There was no elevation of myocardial enzymes.

**Table 1.** Clinical characteristics of 10 neonates with EV infection

Characteristics	No. of cases
Fever>38	3
Rash	3
Diarrhoea	0
Poor feeding	7
Hypotonia	7
Decreased responsiveness	7
Tachycardia	2
Tachypnoea	5
Hepatic injury	1
Myocardial damage	0

Parameters	Mean (range)
Age (days)	26.8 (7–64)
BW (g)	1812.9 (980–3800)
GA (weeks)	31.7 (27.3–39.4)
CRP (mg/dl)	19.5 (0.55–45.96)
Procalcitonin (ng/ml)	0.67 (0.05–4.44)
Leukocytes/mm^3^	9782 (3150–25900)
Cerebrospinal fluid	Normal
Cells/mm^3^	12 (2–96)
Glucose (mg/dL)	2.64 (1.92–3.59)
Protein (mg/dL)	1017 (980–1325)

BW, bodyweight; CRP: C-reactive protein; GA, gestational age.

There were 10 infants with EV infection and 21 infants without EV infection during the epidemic. Clinical data of the two groups were assessed with SPSS 20.0 software. For normally distributed quantitative variables, we reported the mean, standard deviation, and range. For qualitative data, we reported the percentage of the group. Fisher exact test was used, and *P* < 0.05 was considered statistically significant. There were no differences in gestation age, birth weight, delivery mode, use of antibiotics, and parenteral nutrition between the EV group and non-EV group, but the rate of breastfeeding was significantly high in the EV group (Table 2).

**Table 2.** Clinical analysis between the EV group and non-EV group

Risk factor	Cases (*N* = 10) *N* (%)	Non-cases (*N* = 21) *N* (%)	*P*
Caesarean section	3 (30)	6 (28.6)	>0.05
Membrane rupture	2 (20)	4 (19.0)	>0.05
Prematurity	8 (80)	12 (57.1)	>0.05
Hospitalisation (before outbreak)	26.8 19.0	15.8 24.8	>0.05
Breastmilk	8 (80)	5 (23.8)	<0.05
GA (weeks)	31.7±4.0	34.0±4.9	>0.05
Antibiotics (before outbreak)	0 (0)	2 (9.5)	>0.05
PN	1 (10)	2 (9.5)	>0.05
Birth weight	1812.9±1025.1	2330.5±1087.5	>0.05

GA, gestational age; PN, parenteral nutrition.

Emergency reporting to the Medical Service, Nosocomial Infection Department, and Laboratory Department was done. Samples (breastmilk and swabs of body surface and hands, etc.) were collected for bacterial culturing. On 18 July, the Administrative Department of the hospital and the Centers for Disease Prevention and Control were reconvened to assist in investigation and case handling. The affected infants were isolated before treatment start. On the day of onset, patients received 1–2 g/kg gamma globulin and antibiotics until EV positive result. One preterm infant born at a GA of 27 weeks was hospitalised for a long term due to bronchopulmonary dysplasia; the other nine infants were discharged 10 days after onset. Follow-up after discharge showed no abnormality. Upon recovery, all patients were discharged from isolation and symptomatic and supportive treatment.

Further prevention and control measures were initiated against EV infection: (1) Admission of new patients was suspended and receipt of breastmilk was stopped. (2) The offices and care units were disinfected using a chlorinating agent twice a day and then using ultraviolet radiation. (3) The NICU staff washed hands before and after each nursing activity and used disposable gloves (previously rinse-free hand sanitiser was used instead of hand-washing). (4) Recordings of standard operating procedures like changing diapers were provided to all nursing staff (to avoid secondary contamination from excreta). (5) The excreta was sealed using double-layer medical garbage bags (and sprayed with a chlorinated disinfectant on both its inside and outside). (6) Interactions between nursing staff from different work stations of the NICUs were restricted. (7) Breastmilk collection was strictly managed and collection table promptly disinfected after each visit. (8) Telephonic follow-up was done for each discharged patient. Following the above procedure, no new cases of infection were reported.

EV surveillance conducted by the US Centers for Disease Prevention and Control from 2007 to 2008 showed that the most common EV was Coxsackie B1, followed by echovirus types 18, 9, and 6 [4]. The EV infection is commonly transmitted via faecal-oral or oral-oral routes and can also be transplacentally transmitted to the foetus. It has been found to affect neonates much acutely than older children. In a US retrospective study of 783 febrile infants aged under 60 days between 2015 to 2016, 144 (18%) had a positive EV PCR result from any site [5]. A prospective cohort study showed that the positivity of EV RNA detection (stool and CSF samples) in 334 febrile neonates was 39.22% during summer and fall in China [6]. Neonatal EV infection can present sepsis-like manifestations, hepatic injury, myocardial damage, neurological damage, etc. [7, 8]. Different virus serotypes present different clinical manifestations. Clinical severity and outcomes are related to the virus serotype, mode of transmission, and presence of specific antibodies of maternal origin. Hospital-acquired EV infection is associated with lower severity and mortality than that acquired via intrauterine transmission [2]. EVs commonly involve neurological, digestive, and respiratory symptoms, and 70% of severe cases are caused by echovirus 11. An outbreak of echovirus 11 infection in the Cambridge Maternity Hospital was reported in 1977, where three died of the six admitted neonates [9]. The Coxsackie virus commonly presents cardiovascular and neurological symptoms. In China, nosocomial outbreaks of echovirus 11 and Coxsackie virus have been reported in the NICUs, but those of echovirus18 infection in neonates were rare.

A case report has suggested that echovirus 18 can cause sepsis, aseptic meningitis, encephalitis, paralysis, and rashes in neonates and infants [10]. In 1958, echovirus 18 had caused epidemic diarrhoea in 12 neonates and infants aged 6–46 days [11]. In 2008, a Japanese outbreak of echovirus 18 infection in 20 infants was reported in the NICUs, with no poor prognosis in all infants and 55% of them being asymptomatic [3]. However, this outbreak differs from the 2007 echovirus 18 infection reported in Japan, in that victims were predominantly term infants who presented with rash and transient fever. Most of the patients in our NICU were preterm infants, without significant diarrhoea manifestations, but with mild fever, decreased responsiveness, apnoea, and other sepsis-like manifestations, whereas cases involving term infants showed hyperpyrexia, no myocardial damage, and only one case of hepatic injury. There was no sequela after symptomatic and supportive treatment, which is consistent with the case report from Japan. Some reports suggest that neonatal EV infection can cause brain injury [12, 13]. In the present study, cranial MRI for five affected preterm infants revealed no abnormalities, as cranial involvement might predict the activity of specific viral serotypes. CSF samples can more accurately determine whether it is the onset or virus-carrying. Previous studies have shown that an early application of gamma globulin in neonatal EV cases helped reduce mortality [14, 15]. The majority of outbreaks seem to occur in preterm infants. Onset within the first week of birth, echovirus 11, Coxsackie virus, premature birth, male gender, severe hepatitis and multiple organ damage, among others, have been reported to be the risk factors for predicting the severity of EV [16]. Corroborating the fact that EV can be transmitted through breastfeeding [17, 18], eight of the ten cases in our study were breast-fed. Unfortunately, we did not test breastmilk for EV.

The likelihood of an outbreak of EV infection in NICU is much high [19]. It is often difficult to distinguish bacterial from viral infections due to mystifying clinical manifestations. EV testing should be carried out in time for neonates with a negative blood culture, especially for those with fever and rash. Targeted infection prevention and control measures should be undertaken especially during an epidemic season. An outbreak of nosocomial infections in our setting can be attributed to the following reasons: (1) Common wash-free disinfectants are ineffective against EV. The prevention and control of EV emphasises hand-washing, and wash-free disinfectants cannot be a substitute. (2) In the month of July when a new group of nurses were enrolled in the department, training for the prevention and control of nosocomial infections was not in place. (3) All affected neonates were over 7 days of age, thus placental transmission is not justified. On 14 July, there were four cases of onset, among whom the fourth case had to be re-admitted 72 hours after discharge. This infant was on breastfeeding and had been subject to rooming-in before discharge, after which the infant’s father and elder brother also developed fever. Unfortunately, the father and the elder brother were not sampled. A retrospective analysis showed that case 4 could have been the first onset patient and was possibly in the incubation period before discharge. Therefore, the management of rooming-in and expressed breastmilk needs to be strengthened, and visits from parents with suspected intestinal and respiratory tract infections should not be allowed. We didn’t perform tests for the virus on hand swabs of mothers and medical staff, but there was no incidence of fever, diarrhoea, and leave of absence in the staff during the same period. However, we cannot exclude the possibility of the medical staff carrying EV.

## Data Availability

Data supporting the findings of this study are available from the corresponding author upon reasonable request.
